# Isolated Nonspecific Colonic Ulcer: A Case Report From a Tertiary Care Hospital in Pakistan

**DOI:** 10.7759/cureus.5255

**Published:** 2019-07-27

**Authors:** Mehreen K Bhettani, Mubarik Rehman, Humera N Altaf, Syed M Ahmed

**Affiliations:** 1 General Surgery, Royal Oldham Hospital, Manchester, GBR; 2 General Surgery, Shifa College of Medicine, Islamabad, PAK; 3 Surgery, Shifa International Hospital, Islamabad, PAK; 4 General Surgery, University of Maryland Medical Center, Maryland, USA

**Keywords:** pain, ulcer, tachycardia, acute appendicitis, right hemicolectomy

## Abstract

Simple, isolated nonspecific colonic ulcer (NSCU) is a rare pathological entity which has not been adequately explored in the past literature. It is predominant between the ages of 40 and 60 years, and the most common complication is perforation which can be life threatening. Here we present a case of a young female who presented with a perforated NSCU and was successfully managed surgically.

## Introduction

Simple, isolated nonspecific colonic ulcer (NSCU) is a rare pathological entity which has not been adequately explored in the past literature. Although first described in 1832 by Cruveilheir, there are less than 200 reported cases of NSCUs [1]; one of the reasons being that it is a diagnosis of exclusion and many of the cases might have gone unrecognized and undiagnosed [2]. It is predominant between the ages of 40 and 60 years [3], and the most common complication is perforation which can be life threatening [2]. Here we present a case of a young female who presented with a perforated NSCU and was managed surgically.

## Case presentation

A 20-year-old female presented to the emergency department with complaints of pain in the right iliac fossa for the past two days. Pain medications failed to provide any relief, at which point she came to the hospital. She has no history of nausea or vomiting. She also complained of anorexia. The patient did not report any fever or blood in stools. She maintains a balanced healthy diet.

In her past, she had an episode of generalized weakness. On examination she had pallor and on investigation, haemoglobin of 8.2 g/dl was found. She was treated with hematinics by a general practitioner after which her anemia improved. She also has a history of gastric upset with off and on diarrhea and bloating for two years. She has no history of menorrhagia or dysmenorrhea. She is not married and denies the use of cigarette, alcohol, or any illicit drugs.

On examination, the patient was anxious but with tachycardia of 100 beats per minute. There was guarding and tenderness in right iliac fossa with rebound tenderness. Rest of the abdomen was soft and non-tender and none of the viscera was palpable. Psoas and Rovsing signs were positive, whereas Murphy’s and Obturator signs were negative. Bowl sounds were audible and digital rectal examination did not reveal any abnormal finding.

**Figure 1:**
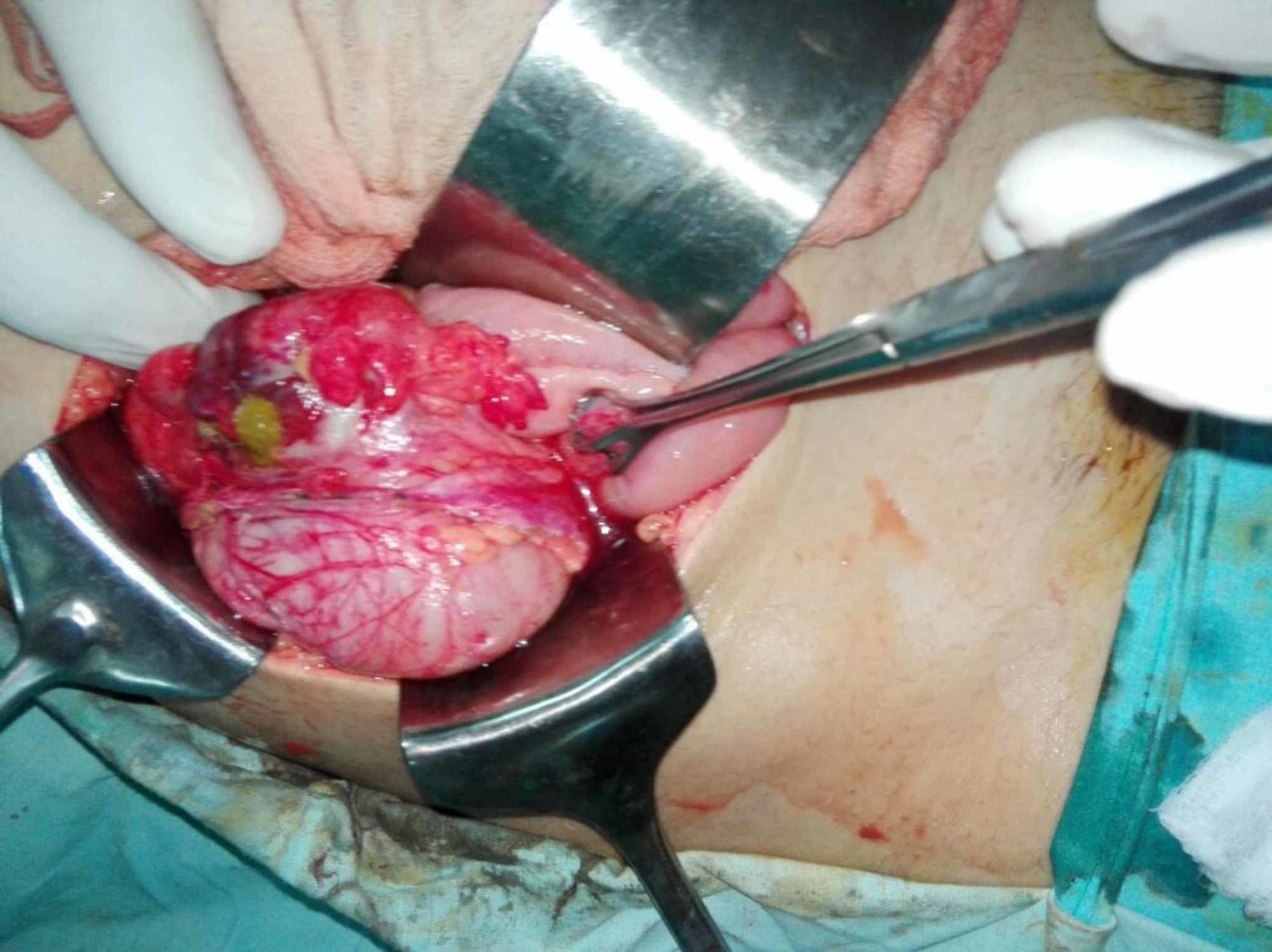
Isolated Nonspecific Colonic Ulcer in the Caecum

Routine investigations were done. Blood complete picture showed total leucocyte count (TLC) of 18700/µl, haemoglobin (Hb) of 9.9 g/dl and neutrophil% of 92.3%. Urine routine examination was normal. The urine pregnancy test was negative. Ultrasonography (USG) abdomen and pelvis was done for ovarian or uterine pathology. USG showed no uterine or ovarian pathology. It showed minimal fluid in the right iliac fossa. Loop of the terminal ileum in right iliac fossa showed paralytic ileus. No normal or abnormal appendix was visualized on USG. Alverado score was 7/10. She was diagnosed as acute appendicitis and was prepared for appendicectomy. Preoperatively, a normal-appearing ileocecal junction and appendix was found which was retrocecal and subserosal. There was a small perforation about 4 cm proximal to the ileocecal junction, measuring approximately 5 mm in diameter on the antimesenteric border with surrounding 2 cm area of cecal wall inflammation as shown in Figure 1. Upon further exploration, mesenteric lymph nodes of the adjacent mesentery were enlarged and firm.

Under suspicion of malignancy, right limited hemicolectomy was performed. The specimen was sent for histopathological examination. The histopathology reported extensive mucosal ulcerations, transmural inflammation, and abscess formation along with serositis. However, no granuloma, infective organism, or malignancy was noted. The lymph nodes also showed signs of benign reactive changes.

Her post-operative course was uneventful and she was discharged after three days of her surgery with instructions to follow up after two weeks or earlier. Two weeks later, per stoma proximal and distal colonoscopy was performed that did not show any abnormality. Ileocolostomy reversal was performed after one month. The patient was followed up for six months during which period she remained healthy.

## Discussion

NSCUs have been referred by various terminologies in the previous studies such as idiopathic ulcers, cecal ulcers, simple ulcers of the colon, solitary ulcers, idiopathic ulcers, among others [1]. However, there is limited knowledge with regards to its etiology and still remains a diagnosis of exclusion [4]. 

NSCUs can affect all age groups; however, it is most common in the fifth decade [3] with a slight female predominance [3-4]. According to Ong et al., 67% of the ulcers occur in the cecum and the ascending colon [3], with the majority developing in the cecum near the ileocecal valve [2]. They usually occur as solitary lesions on the antimesenteric border on the luminal side of the colon, and may be round or oval but with sharply demarcated borders surrounded by normal colonic mucosa [4-5]. Their size and depth may vary from a few millimeters to centimeters, and from abrasions to full depth perforations [2].

To date its etiology is unknown. However, numerous theories have been put forth in past literature, but none has been substantiated. According to the ischemic hypothesis, vascular abnormalities such as vascular wall thickening and thrombus formation in microvasculature can lead to vascular insufficiency resulting in the ulcer formation. This has been noted in the area surrounding the ulcer and could explain its location on the antimesenteric border, however, if ischemia were to be the cause then the majority of the ulcers would have developed in the watershed areas (descending colon and the sigmoid colon) rather than in the cecum [4].

Another proposed mechanism lists diverticulosis as the potential cause as cecal diverticula have been observed in cases with NSCUs. Again, the facts that go against this theory are that cecal diverticula usually form on the mesenteric border (rather than the antimesenteric border as is the case with NSCUs) and furthermore are prevalent in the sigmoid colon as opposed to the cecum [4]. 

Other theories put forth regarding the causal factors contributing to the development of NSCUs includes a change in the pH of the digestive contents in the ileocecal region affecting the colonic mucosa, overuse of drugs including nonsteroidal anti-inflammatory drugs, oral contraceptives, and corticosteroids, colonic trauma caused by foreign body ingestion, and infections and toxins [4,6-8].

Signs and symptoms depend on the stage of presentation. In the acute phase, the most common manifestation is right lower quadrant pain which might follow epigastric pain [2], mimicking acute appendicitis. In fact, Ong et al. estimate this clinical feature to be present in 50% of the cases [3], whereas Lazarovitch et al., while reviewing 80 cases of NSCUs, found that in 68% of the cases the initial diagnosis made was acute appendicitis [9]. Nausea and vomiting may or may not be present, but mild leukocytosis and fever usually is. On examination, localized tenderness is present, along with muscle rigidity and, sometimes, rebound tenderness [2]. The most common complication during this phase is gut perforation, in which case signs of acute peritonitis will be present and free air under the diaphragm would be radiologically demonstrable [2]. Lazarovitch et al. estimated that 66% of the NSCUs on the right and 81% of the ulcers on the left side of the colon were already perforated at the time of presentation [9]. Constipation occurs in most of the cases [2], while lower gastrointestinal bleeding was shown to be present in 33% of the cases [3], however, melena is rare [2].

In subacute or chronic cases, the patient usually presents with a palpable mass in the right lower quadrant along with low grade vague abdominal pain, which was in many cases confused with malignancy resulting in right hemicolectomy [2,4]. If healing of the ulcer has already occurred, the presentation could be of bowel obstruction secondary to cicatrization [2].

The test of choice for diagnosis is colonoscopy with multiple biopsies which usually show nonspecific signs of both acute and chronic inflammation [2-4]. Therefore, other conditions of the colon which could cause ulcerations should be ruled out to reach a diagnosis of NSCU. 

With regards to the treatment, uncomplicated ulcers should be managed conservatively with colonoscopy follow-up every 4-6 weeks and each time multiple biopsies should be taken to ensure healing, and exclude malignancy and infection [3-4]. For complicated ulcers, such as those cases which have progressed to bleeding, perforation, abscess formation, or which have failed to heal on endoscopic examination, should be surgically managed [4], as studies have shown that conservative management of cases with perforated NSCUs has a high mortality rate [9-10]. The surgical options include localized excision or oversewing of the ulcer, or right hemicolectomy [4].

## Conclusions

Our case demonstrates that even though NSCUs are most prevalent in the fifth decade, they can occur at any age and surgeons should be mindful of this condition when dealing with cases of acute appendicitis in young individuals, so that these cases do not go undiagnosed which could result in fatal consequences. We have also shown that right hemicolectomy is a suitable treatment option for perforated NSCUs. The pathogenesis of NSCUs still eludes us and much research should be focused on its etiology, because only then will we be able to manage it more successfully. 
